# Burnout Dimensions and Job Satisfaction Among Critical Care Nurses at a Regional Hospital in Oman: A Cross‐Sectional Analysis of Work‐Related Contributing Factors

**DOI:** 10.1155/jonm/5186923

**Published:** 2026-09-07

**Authors:** Rawya Al Shereiqi, Yousef Al Seraei

**Affiliations:** ^1^ Department of Adult Intensive Care Unit, Rustaq Hospital, Al Rustaq, South Al Batinah Governorate, Oman

**Keywords:** burnout, critical care unit, job satisfaction, nurses, Oman

## Abstract

**Background:**

Burnout and job satisfaction are major concerns among critical care nurses, yet evidence from Oman’s regional hospitals remains limited. This study examined the relationship between burnout and job satisfaction among critical care nurses.

**Method:**

A cross‐sectional correlational design was used, with a stratified sample of 150 critical care nurses across five units at an Omani regional hospital. Data were collected using the Maslach Burnout Inventory‐Human Services Survey for Medical Personnel (MBI‐HSS (MP)) and the job satisfaction survey (JSS) and were analyzed using SPSS v31.

**Results:**

Participants reported moderate burnout (*M* = 2.90, SD = 0.78) and ambivalent job satisfaction (*M* = 3.62, SD = 0.57). Burnout varied by unit, with higher emotional exhaustion and depersonalization in the neonatal intensive care unit and the emergency department. Burnout was negatively correlated with job satisfaction (*r = *−0.40, *p* < 0.001). Regression indicated that the nurse‐to‐patient ratio was associated with burnout, whereas nationality was associated with job satisfaction. Emotional exhaustion fully mediated the link between the nurse‐to‐patient ratio and job satisfaction (indirect β = −0.12, Sobel *z* = −3.44, *p* < 0.001).

**Conclusion:**

Workload drivers directly exacerbate burnout and reduce job satisfaction among critical care nurses. It is recommended that strategies be implemented to improve staffing levels, provide supportive leadership, and promote professional development to enhance nurse well‐being and retention.

**Implications for Nursing Management:**

Nurse managers can use the findings of this paper to guide tiered staffing during high‐demand shifts, strengthen peer‐support and debriefing protocols, expand promotion and professional development pathways, and design retention strategies that address the differing needs of local and foreign nurses, focusing on workload controls and career progression for local nurses and on contractual stability and mentorship for expatriate staff.

## 1. Introduction

Nursing is widely recognized as a demanding profession, particularly in critical care settings where nurses manage acutely ill patients, heavy workloads, and emotionally intense environments. Compared with other hospital units, critical care nurses provide complex, high‐risk care [1]. Consequently, sustained exposure to these demands may lead to occupational burnout, which correlates with higher turnover and job dissatisfaction [1]. Despite growing global attention to nurse well‐being, evidence regarding critical care nurses in Oman remains limited, particularly in secondary‐level hospitals. This study addresses this gap by examining burnout and job satisfaction in this under‐researched context.

Burnout is a significant occupational concern in healthcare, defined by the World Health Organization (WHO) [2] as a syndrome resulting from unmanaged workplace stress, characterized by emotional exhaustion (EE), depersonalization (DP), and reduced personal accomplishment (PA). Conversely, job satisfaction reflects the fulfillment, positive emotional response, and perceived equity experienced within the work environment [3]. International studies report moderate burnout levels among nurses [4], with similar trends across the Middle East [5]. Evidence from Oman indicates comparable findings, showing moderate burnout in tertiary hospitals [6] and moderate job satisfaction in regional settings [7]. Higher burnout is consistently associated with lower job satisfaction. Nurses experiencing high burnout frequently report emotional distress, lower work engagement, and turnover intention [8]. In contrast, higher job satisfaction correlates with stronger professional commitment, superior performance, and improved patient outcomes, whereas dissatisfaction increases absenteeism and reduces care quality [9].

Although widely studied internationally, Omani research has predominantly focused on tertiary‐level hospitals, leaving secondary‐level hospitals—where severe stressors coexist with fewer institutional resources—under‐examined. Additionally, few studies examine burnout and job satisfaction simultaneously or explore associated demographic and workplace predictors in regional critical care units. Addressing these gaps is essential for workforce planning. This study examined the relationship between burnout and job satisfaction among critical care nurses in a regional hospital in Oman while identifying key demographic and workplace factors. We hypothesized that higher burnout correlates with lower job satisfaction (Hypothesis 1) and that EE exhibits a pattern consistent with statistical mediation between nurse‐to‐patient ratios and job satisfaction (Hypothesis 2).

This study was guided by conservation of resources (COR) theory [10], which posits that individuals strive to acquire, retain, and protect valued resources such as energy, emotional stability, and time. According to COR theory, burnout develops when these resources are threatened or depleted. In critical care, high nurse‐to‐patient ratios drain time and energy, triggering EE. As resource losses accumulate, loss spirals develop [11], leading to DP, reduced accomplishment, and diminished job satisfaction. This framework directly underpins Hypothesis 2. By identifying key workplace determinants, these findings provide nurse leaders and administrators with actionable insights to strengthen work environments, enhance staff retention, and support healthcare sustainability.

## 2. Methods

### 2.1. Study Design

A quantitative cross‐sectional correlational design was used to examine the relationship between burnout and job satisfaction among critical care nurses. This design was selected to evaluate relationships among variables at a single point in time.

### 2.2. Setting

The study was conducted at a regional secondary healthcare facility in Batinah South, Oman, between June and August 2025. The facility includes five critical care units: adult intensive care units (AICUs), pediatric intensive care units (PICUs), neonatal intensive care units (NICUs), operating theater (OT), and emergency departments (EDs).

### 2.3. Participants and Sampling

A proportionate stratified random sampling method was used to ensure proportional representation across the five critical care units. Based on a total target sample size of 152 nurses, 150 completed the survey, yielding a 98.7% response rate. Inclusion criteria required nurses to have at least 6 months of full‐time critical care experience. Exclusion criteria comprised nursing students, trainees, general ward nurses, and those on extended leave.

### 2.4. Data Collection

Data were collected via a secure Google Form distributed to eligible nurses from June to August 2025 through official unit communication channels. An introductory page detailed study objectives, procedures, confidentiality, and voluntary consent. A reminder message was issued 1 week following initial distribution to maximize participation.

### 2.5. Instruments

#### 2.5.1. Burnout

Burnout: Burnout was assessed using the Maslach Burnout Inventory‐Human Services Survey for Medical Personnel (MBI‐HSS (MP)). The 22‐item instrument utilizes a 7‐point Likert scale (0 = “never” to 6 = “every day”) across three subscales: EE, DP, and PA. Higher EE and DP scores, coupled with lower PA scores, indicate higher burnout. Standard cutoff values were used to classify burnout levels. The instrument demonstrated strong reliability (*α* = 0.71–0.90) [12]. Usage permission was secured via Mind Garden.

### 2.6. Job Satisfaction

Job satisfaction: Job satisfaction was assessed using the job satisfaction survey (JSS), a 36‐item instrument evaluating overall satisfaction across nine subscales: pay, promotion, supervision, fringe benefits, contingent rewards, operating procedures, coworkers, nature of work, and communication [13]. Items are rated on a six‐point Likert scale, with negative items reverse‐scored. Total mean scores reflect the overall satisfaction, categorized as dissatisfied (1.00–2.99), ambivalent (3.00–3.99), or satisfied (4.00–6.00). The JSS demonstrated high reliability (*α* = 0.91) [13]. Permission was obtained from the author.

### 2.7. Ethical Procedures

Ethical approval was obtained from the Research and Ethical Review & Approval Committee in Batinah South (SBG) under the Ministry of Health (MOH) and the National Center of Health Research (Research code: 03072025, Date: July 20, 2025). The research complied with the ethical standards set by the Research and Ethical Review & Approval Committee, which are governed by a combination of national regulatory guidelines and the universal principles of the Declaration of Helsinki and WHO standards. Additionally, nurse participation in the study was voluntary, anonymous, and kept highly confidential. Invitations to participate were sent through social media platforms. They included details on the purpose, methods, duration, and data‐collection procedures, as well as how the data would be used and any potential risks or discomfort associated with the study. Participants were asked to sign electronic informed consent before completing the Google Forms questionnaire, with a strong emphasis on voluntary, anonymous participation.

### 2.8. Statistical Analysis

Data were analyzed using IBM SPSS Statistics software, version 31. Descriptive statistics, including means, standard deviations, frequencies, and percentages, summarized demographic variables and measured levels of burnout and job satisfaction. Inferential analyses examined differences and relationships among study variables. Independent‐samples t‐tests and one‐way between‐subjects ANOVAs were used to compare burnout and job satisfaction subscale scores across demographic and work‐related factors. Bivariate Pearson correlation coefficients (*r*) were used to assess the relationships between burnout (EE, DP, and PA) and job satisfaction subscales. Multiple regression analyses identified predictors of burnout and job satisfaction among critical care nurses. In addition, a mediation analysis was conducted to examine whether EE mediated the association between the nurse‐to‐patient ratio and job satisfaction. This examined whether EE mediated the indirect relationship between nurse‐to‐patient ratios (predictor) and job satisfaction (outcome). For all inferential tests, a two‐tailed *p*‐value less than 0.05 indicated statistical significance. Effect sizes (e.g., *r* ^2^, eta^2^) were calculated to complement significance testing and indicate the strength of observed relationships.

## 3. Results

### 3.1. Participant Flow and Demographic Characteristics

A total of 150 critical care nurses participated (98.7% response rate). Most were female (80.0%), Omani nationals (83.3%), and married (71.3%). Participants were primarily assigned to adult intensive care and EDs, with smaller numbers working in OTs and in NICUs and PICUs. Most nurses held a bachelor’s degree (60.7%) and worked rotating shifts. The mean age was 31.8 years (SD = 6.15), and the mean clinical experience was 8.4 years (SD = 6.91). Approximately one‐fifth (20.7%) reported a health condition. Most nurses worked at nurse‐to‐patient ratios of 1:1 or ≥ 1:3, and more than half (53.3%) reported being occasionally required to perform unscheduled duties (Supporting Table S1).

### 3.2. Reliability

Internal consistency was acceptable to excellent for the MBI‐HSS (MP) subscales: EE (*α* = 0.87), DP (*α* = 0.64), and PA (*α* = 0.78). The JSS also demonstrated excellent reliability (*α* = 0.86).

### 3.3. Burnout and Job Satisfaction Levels

Nurses reported moderate EE (*M* = 2.71, SD = 1.36) and DP (*M* = 1.64, SD = 1.21), with moderate PA (*M* = 4.35, SD = 1.08). Overall, burnout was moderate (*M* = 2.90, SD = 0.78; Supporting Table S2). Job satisfaction was ambivalent (*M* = 3.62, SD = 0.57). Satisfaction was highest for the nature of work (*M* = 4.73, SD = 0.92) and supervision (*M* = 4.26, SD = 1.00) and lowest for promotion (*M* = 3.09, SD = 1.02) and fringe benefits (*M* = 3.03, SD = 0.92; Supporting Table S3).

### 3.4. Relationship Between Burnout and Job Satisfaction

EE was positively correlated with DP (*r* = 00.59, *p* < 0.001) and overall burnout (*r* = 00.81, *p* < 0.001) and negatively correlated with PA (*r* = −0.17, *p* = 0.043) and job satisfaction (*r* = −0.64, *p* < 0.001). DP was positively associated with overall burnout (*r* = 0.78, *p* < 0.001) and negatively associated with job satisfaction (*r* = −0.32, *p* < 0.001). PA was positively correlated with job satisfaction (*r* = 0.28, *p* < 0.001). Overall, burnout was negatively correlated with job satisfaction (*r* = −0.40, *p* < 0.001; Table 1).

**TABLE 1:** Pearson correlation coefficients (*r*) among burnout subscales and job satisfaction (*N* = 150).

Variable	1	2	3	4	5
1. EE	—				
2. DP	0.59^∗∗∗^	—			
3. PA	−0.17^∗^	−0.17^∗^	—		
4. Total Burnout	0.81^∗∗∗^	0.78^∗∗∗^	0.28^∗∗∗^	—	
5. JSS	−0.64^∗∗∗^	−0.32^∗∗∗^	0.28^∗∗∗^	−0.40^∗∗∗^	—

Abbreviations: DP = depersonalization, EE = emotional exhaustion, JSS = job satisfaction survey, PA = personal accomplishment.

^∗^ *p* < 0.05.

^∗∗^ *p* < 0.01.

^∗∗∗^ *p* < 0.001.

### 3.5. Contributing Factors for Burnout and Job Satisfaction (t‐Test)

No significant differences were observed between male and female nurses in EE, DP, PA, overall burnout, or job satisfaction (all *p* > 0.05). By nationality, Omani nurses reported higher EE (*t* = 9.10, *p* < 0.001) and DP (*t* = 3.17, *p* = 0.002) and greater overall burnout (*t* = 5.90, *p* < 0.001) than non‐Omani nurses. In contrast, non‐Omani nurses reported higher PA (*t* = −2.86, *p* = 0.005) and better job satisfaction (*t* = −7.93, *p* < 0.001). Nurses reporting health problems had higher EE (*t* = 2.75, *p* = 0.004) and overall burnout (*t* = 2.01, *p* = 0.046) than those without health problems, whereas no differences were observed in DP, PA, or job satisfaction (all *p* > 0.05). Work schedule was not associated with EE, DP, PA, overall burnout, or job satisfaction (all *p* > 0.05; Table 2).

**TABLE 2:** Independent t‐test results for burnout and job satisfaction across contributing factors (*N* = 150).

Subscale	Category (*n*)	*M* (SD)	*t* (*df*)	*p*	Cohen’s d
*Gende*r					
EE	Male (30) Female (120)	2.71 (1.34) 2.71 (1.37)	−0.02 (148)	0.984	−0.004
DP	Male (30) Female (120)	1.74 (1.26) 1.61 (1.19)	0.52 (148)	0.604	0.12
PA	Male (30) Female (120)	4.61 (0.99) 4.28 (1.09)	1.47 (148)	0.143	0.30
Total Burnout	Male (30) Female (120)	3.02 (0.70) 2.87 (0.80)	0.94 (148)	0.350	0.19
JSS	Male (30) Female (120)	3.46 (0.58) 3.66 (0.56)	−1.75 (148)	0.082	−0.36

*Nationality*					
EE	Omani (125) Non‐Omani (25)	3.02 (1.23) 1.18 (0.84)	9.10 (47.25)	< 0.001	1.56
DP	Omani (125) Non‐Omani (25)	1.77 (1.21) 0.96 (0.94)	3.17 (148)	0.002	0.70
PA	Omani (125) Non‐Omani (25)	4.23 (1.05) 4.90 (1.04)	−2.86 (148)	0.005	−0.63
Total Burnout	Omani (125) Non‐Omani (25)	3.01 (0.79) 2.35 (0.44)	5.90 (60.52)	< 0.001	0.90
JSS	Omani (125) Non‐Omani (25)	3.48 (0.47) 4.31 (0.50)	−7.93 (148)	< 0.001	−1.74

*Health Problems*					
EE	Yes (31) No (119)	3.30 (1.19) 2.56 (1.37)	2.75 (148)	0.004	0.57
DP	Yes (31) No (119)	1.66 (1.23) 1.63 (1.21)	0.14 (148)	0.888	0.03
PA	Yes (31) No (119)	4.48 (0.97) 4.32 (1.10)	0.76 (148)	0.452	0.15
Total Burnout	Yes (31) No (119)	3.15 (0.77) 2.83 (0.77)	2.01 (148)	0.046	0.41
JSS	Yes (31) No (119)	3.46 (0.51) 3.66 (0.57)	−1.71 (148)	0.073	−0.35

*Work Schedule*					
EE	Standard (11) Shift (139)	3.10 (1.17) 2.68 (1.37)	0.99 (148)	0.325	0.40
DP	Standard (11) Shift (139)	1.59 (1.58) 1.64 (1.17)	−0.16 (148)	0.875	−0.50
PA	Standard (11) Shift (139)	4.65 (0.79) 4.33 (1.10)	0.95 (148)	0.343	0.30
Total Burnout	Standard (11) Shift (139)	3.11 (0.92) 2.88 (0.77)	0.93 (148)	0.353	0.29
JSS	Standard (11) Shift (139)	3.38 (0.46) 3.63 (0.57)	−1.45 (148)	0.149	−0.45

Abbreviations: DP = depersonalization, EE = emotional exhaustion, JSS = job satisfaction survey, M = mean, PA = personal accomplishment, SD = standard deviation.

#### 3.5.1. Contributing Factors for Burnout and Job Satisfaction (One‐Way ANOVA)

**FIGURE 1:**
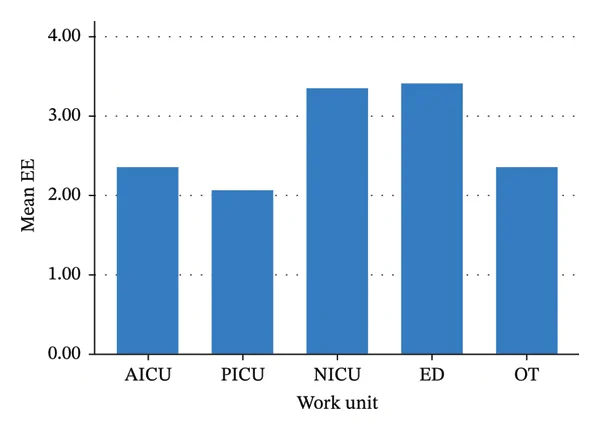
Mean levels of emotional exhaustion (EE) among nurses working in different hospital units. Note. AICU = adult intensive care unit; PICU = pediatric intensive care unit; NICU = neonatal intensive care unit; ED = emergency department; OT = operating theater.

No significant differences were observed by educational level or marital status across EE, DP, PA, overall burnout, or job satisfaction (all *p* > 0.05). Burnout differed significantly by work unit for EE (*F* = 5.79, *p* < 0.001, *η* ^2^ = 0.14), DP (*F* = 6.16, *p* < 0.001, *η* ^2^ = 0.15), overall burnout (*F* = 4.70, *p* = 0.001, *η* ^2^ = 0.12), and job satisfaction (*F* = 3.04, *p* = 0.019, *η* ^2^ = 0.08) but not for PA (*F* = 1.36, *p* = 0.25). Post hoc comparisons showed that nurses in emergency and NICUs reported higher EE and overall burnout than those in OTs and AICUs (Figure 1). DP was highest among emergency nurses, whereas job satisfaction was highest among those working in AICUs.

**FIGURE 2:**
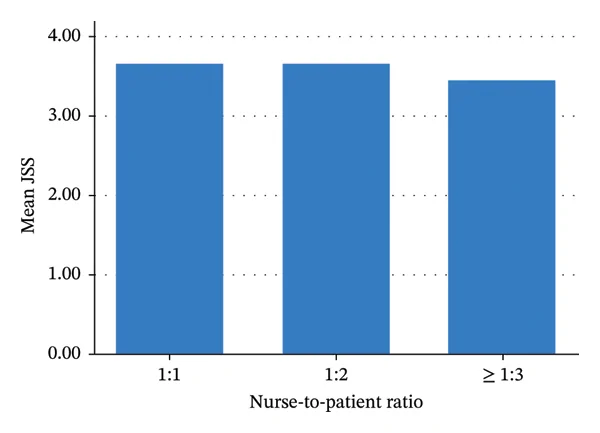
Mean job satisfaction (JSS) scores based on nurse‐to‐patient ratios.

Nurse‐to‐patient ratio was significantly associated with EE (*F* = 6.93, *p* = 0.001, *η* ^2^ = 0.09), overall burnout (*F* = 4.43, *p* = 0.014, *η* ^2^ = 0.06), and job satisfaction (*F* = 5.33, *p* = 0.006, *η* ^2^ = 0.07) but not with DP or PA (both *p* > 0.05). Post hoc comparisons indicated that nurses responsible for three or more patients reported higher EE and overall burnout than those caring for one patient. Job satisfaction was lower among nurses assigned three or more patients than among those with 1:1 or 1:2 assignments (Figure 2).

No significant differences were observed across the frequency of unscheduled duty requests for EE, DP, PA, overall burnout, or job satisfaction (all *p* > 0.05; Table 3). Effect sizes were small across all outcomes (*η* ^2^ = 0.02–0.05). Although nurses who were more frequently requested to attend unscheduled duty reported higher mean burnout scores, these differences were not statistically significant.

**TABLE 3:** One‐way ANOVA results for burnout dimensions and job satisfaction across contributing factors (*N* = 150).

Factors	Subscale	*df*	*F*	*p*	*η* ^2^
Educational Level	EE	(2, 147)	0.24	0.788	0.003
	DP	(2, 147)	1.55	0.216	0.021
	PA	(2, 147)	1.72	0.183	0.023
	Total Burnout	(2, 147)	0.17	0.841	0.002
	JSS	(2, 147)	0.24	0.784	0.003

Marital Status	EE	(2, 147)	0.61	0.547	0.008
	DP	(2, 147)	0.33	0.720	0.004
	PA	(2, 147)	1.45	0.239	0.019
	Total Burnout	(2, 147)	1.01	0.367	0.014
	JSS	(2, 147)	2.34	0.100	0.031

Work Unit	EE	(4, 145)	5.79	< 0.001	0.14
	DP	(4, 145)	6.16	< 0.001	0.15
	PA	(4, 145)	1.36	0.25	0.04
	Total Burnout	(4, 145)	4.70	< 0.001	0.12
	JSS	(4, 145)	3.04	0.019	0.08

Nurse‐to‐Patient Ratio	EE	(2, 147)	6.93	< 0.001	0.09
	DP	(2, 147)	1.16	0.316	0.02
	PA	(2, 147)	0.59	0.555	0.01
	Total Burnout	(2, 147)	4.43	0.014	0.06
	JSS	(2, 147)	5.33	0.006	0.07

Unscheduled Duty Requests	EE	(4, 145)	0.83	0.508	0.02
	DP	(4, 145)	1.33	0.263	0.04
	PA	(4, 145)	1.98	0.101	0.05
	Total Burnout	(4, 145)	1.91	0.111	0.05
	JSS	(4, 145)	0.85	0.496	0.02

*Note:* *η* ^2^ = eta‐squared effect size.

Abbreviations: DP, depersonalization; EE, emotional exhaustion; JSS, job satisfaction survey; PA, personal accomplishment.

Age was negatively correlated with EE (*r* = −0.19, *p* = 0.018) and DP (*r* = −0.25, *p* = 0.002) and positively correlated with PA (*r* = 0.21, *p* = 0.009). No significant associations were observed between age and overall burnout or job satisfaction (both *p* > 0.05). Years of experience showed a similar pattern, with negative correlations with DP (*r* = −0.21, *p* = 0.008) and positive correlations with PA (*r* = 0.23, *p* = 0.005), but no associations with EE, overall burnout, or job satisfaction (all *p* > 0.05; Table 4).

**TABLE 4:** Pearson’s correlation coefficients (*r*) between age, years of experience, burnout dimensions, and job satisfaction (*N* = 150).

Factors	EE	DP	PA	Total burnout	JSS
Age	−0.19^∗^	−0.25^∗∗^	0.21^∗∗^	−0.14	0.12
Years of Experience	−0.12	−0.21^∗∗^	0.23^∗∗^	−0.07	0.03

Abbreviations: DP = depersonalization, EE = emotional exhaustion, JSS = job satisfaction survey, PA = personal accomplishment.

^∗^ *p* < 0.05 *.*

^∗∗^ *p* < 0.01.

### 3.6. Multiple Regression Analysis

Multicollinearity was identified between age and years of experience (*r* = 0.95); therefore, years of experience was excluded from the final models. All remaining predictors met regression assumptions (tolerance > 0.20; VIF < 3.5). The burnout model was significant (*F* (10,139) = 3.36, *p* < 0.001) and explained 20% of the variance (*R* ^2^ = 0.20). Nationality (β = −0.23, *p* = 0.040) and nurse‐to‐patient ratio (β = 0.23, *p* = 0.012) were the only significant predictors (Table 5). The job satisfaction model was also significant (*F* (10,139) = 7.66, *p* < 0.001), accounting for 36% of the variance (*R* ^2^ = 0.36). Nationality was the only significant predictor (β = 0.59, *p* < 0.001), with all other variables nonsignificant (Table 6). A summary of significant predictors is presented in Table 7.

**TABLE 5:** Multiple linear regression predicting burnout among critical care nurses (*N* = 150).

Predictor	*B* (SE)	β	*t*	*p*
Constant	4.395 (1.192)	—	3.688	< 0.001
Gender	−0.134 (0.158)	−0.069	−0.851	0.396
Age	−0.013 (0.018)	−0.105	−0.752	0.453
Nationality	−0.470 (0.226)	−0.226	−2.076	0.040^∗^
Education level	−0.102 (0.149)	−0.071	−0.684	0.495
Marital status	0.054 (0.138)	0.034	0.387	0.699
Health problems	−0.264 (0.159)	−0.138	−1.662	0.099
Work unit	−0.049 (0.046)	−0.097	−1.063	0.289
Nurse‐to‐patient ratio	0.207 (0.081)	0.228	2.556	0.012^∗^
Work schedule	−0.197 (0.270)	−0.066	−0.730	0.466
Unscheduled duty Requests	0.138 (0.076)	0.141	1.812	0.072

*Note:* B = unstandardized coefficients; β = standardized coefficients. *R* ^2^ = 0.195, adjusted *R* ^2^ = 0.137, F (10,139) = 3.36, *p* < 0.001.

Abbreviation: SE = standard errors.

^∗^ *p* < 0.05.

^∗∗^ *p* < 0.01.

^∗∗∗^ *p* < 0.001.

**TABLE 6:** Multiple linear regression predicting job satisfaction among critical care nurses (*N* = 150).

Predictor	*B* (SE)	β	*t*	*p*
Constant	3.625 (0.775)	—	4.674	< 0.001
Gender	0.086 (0.102)	0.061	0.837	0.404
Age	−0.017 (0.011)	−0.184	−1.479	0.141
Nationality	0.886 (0.147)	0.585	6.019	< 0.001^∗∗∗^
Education level	−0.095 (0.097)	−0.092	−0.983	0.327
Marital status	−0.006 (0.090)	−0.005	−0.068	0.946
Health problems	0.037 (0.104)	0.027	0.361	0.718
Work unit	−0.014 (0.030)	−0.037	−0.457	0.648
Nurse‐to‐patient ratio	−0.100 (0.053)	−0.152	−1.910	0.058
Work schedule	−0.068 (0.175)	−0.032	−0.389	0.698
Unscheduled duty requests	−0.065 (0.050)	−0.091	−1.310	0.192

*Note:* B = unstandardized coefficients; β = standardized coefficients. *R* ^2^ = 0.355, adjusted *R* ^2^ = 0.309, *F* (10,139) = 7.66, *p* < 0.001.

Abbreviation: SE = standard errors.

^∗^ *p* < 0.05.

^∗∗^ *p* < 0.01.

^∗∗∗^ *p* < 0.001.

**TABLE 7:** Significant predictors of burnout and job satisfaction among critical care nurses (*N* = 150).

Dependent variable	Predictor	*B* (β)	*p*
Burnout	Nationality	−0.47 (−0.23)	0.040^∗^
	Nurse‐to‐patient ratio	0.21 (0.23)	0.012^∗^

Model fit	*R* ^2^ = 0.20, Adjusted *R* ^2^ = 0.14, *F* (10, 139) = 3.36, *p* < 0.001		

Job Satisfaction	Nationality	0.89 (0.59)	< 0.001^∗∗∗^

Model fit	*R* ^2^ = 0.36, Adjusted *R* ^2^ = 0.31, *F* (10, 139) = 7.66, *p* < 0.001		

*Note:* B = unstandardized coefficients; β = standardized coefficients.

^∗^ *p* < 0.05.

^∗∗∗^ *p* < 0.001.

### 3.7. Mediation Analysis

To test the COR‐derived hypothesis that EE mediates the pathway from nurse‐to‐patient ratio to job satisfaction, a four‐step mediation analysis was conducted [14]. The mediator was the mean score of the nine items of the EE subscale, the predictor was the nurse‐to‐patient ratio, coded as 1 = 1:1, 2 = 1:2, 3 = ≥ 1:3, and the outcome was the mean score of the JSS. The analysis was based on a sample of 150 participants. In step 1, the nurse‐to‐patient ratio was significantly and negatively associated with job satisfaction (*β* = −0.23, *t* = −2.90, *p* = 0.004; c path). In step 2, the ratio was significantly associated with EE (β = 0.29, *t* = 3.70, *p* < 0.001; a path). In step 3, EE was significantly and negatively associated with job satisfaction, controlling for ratio (β = −0.62, *t* = −9.32, *p* < 0.001; b path). Finally, in step 4, the direct effect of ratio on job satisfaction became nonsignificant when EE was included (β = −0.05, *t* = −0.77, *p* = 0.442; c’ path), while EE remained significant. The indirect effect was statistically significant (indirect β = −0.12, Sobel *z* = −3.44, *p* < 0.001 [15]), supporting a statistically significant full mediation model (Table 8).

**TABLE 8:** Mediation analysis: emotional exhaustion as a mediator between nurse‐to‐patient ratio and job satisfaction (*N* = 150).

Step	Path	β	*t*	*p*	Interpretation
1 – c (total)	Ratio ⟶ JSS	−0.23	−2.90	0.004	Significant total effect
2 – a path	Ratio ⟶ EE	0.29	3.70	< 0.001	Ratio associated with EE
3 – b path	EE ⟶ JSS | Ratio	−0.62	−9.32	< 0.001	EE associated with JS
4 – c’ (direct)	Ratio ⟶ JSS | EE	−0.05	−0.77	0.442	Nonsignificant direct effect
Indirect	Ratio ⟶ EE ⟶ JSS	−0.12	*Sobel* *z* = −3.44	< 0.001	Pattern consistent with full mediation

Abbreviations: EE = emotional exhaustion, JSS = job satisfaction survey.

#### 3.7.1. Exploratory Analyses (Chi‐Square)

A significant association was found between work unit and nurse‐to‐patient ratio, *χ* ^2^ (8, *N* = 150) = 72.52, *p* < 0.001. Nurses working in the NICU (73.1%) and ED (57.1%) were more likely to be responsible for three or more patients than those in the AICU and PICU, who predominantly had 1:1 assignments (see Supporting Table S4).

## 4. Discussion

The study evaluated the hypothesized relationships and empirical pathways among critical care nurses in a regional hospital setting. Consistent with Hypothesis 1, higher burnout was significantly associated with lower job satisfaction, with all three burnout dimensions exhibiting moderate to strong negative correlations (*r* = −0.63 to −0.31, *p* < 0.001). Furthermore, supporting Hypothesis 2, EE mediated the relationship between nurse‐to‐patient ratios and job satisfaction. Descriptive analyses showed moderate burnout levels across all MBI‐HSS (MP) dimensions (EE *M* = 2.71, DP *M* = 1.64, PA *M* = 4.35) according to established cutoff thresholds [12]. Overall job satisfaction was also moderate (*M* = 3.62), reflecting ambivalent work environment perceptions. Collectively, these findings indicate that critical care nurses in this regional hospital experience moderate occupational strain, highlighting a critical window for preventive organizational interventions.

These findings suggest that, despite significant emotional stress, many nurses retain professional fulfillment. These results align with global evidence showing a moderate prevalence of burnout among nurses in high‐stress units. Meta‐analytic and multisite studies estimate that 31%–50% of ICU nurses experience high burnout [16, 17]. Developing nations report similar moderate‐to‐high burnout driven by heavy workloads and staffing shortages [18, 19]. Similarly, in Oman, Qutishat and Al Sabei [6] observed moderate burnout among tertiary critical care nurses (*M* = 38.93, SD = 8.6). Although measurement tools and reporting formats vary across studies, overall evidence consistently demonstrates moderate burnout levels, underscoring the ongoing need for targeted workplace interventions in Omani healthcare settings.

Despite high demand, nurses reported ambivalent overall job satisfaction (*M = *3.62, SD *= *0.57). Subscale scores were highest for the nature of work (*M* = 4.73, SD = 0.92) and supervision (*M* = 4.26, SD = 1.00) and lowest for promotion (*M* = 3.09, SD = 1.02) and fringe benefits (*M* = 3.03, SD = 0.92). These results indicate that nurses remain dissatisfied with extrinsic factors such as advancement opportunities and compensation, yet value intrinsic aspects like care delivery and supportive leadership. International studies mirror these trends, with nurses reporting greater satisfaction with professional and interpersonal domains than organizational policies [20–23]. High satisfaction with the nature of work and supervision aligns with evidence that supportive leadership and intrinsic motivators drive retention [23, 24]. Conversely, dissatisfaction with promotion and financial incentives reflects widespread patterns observed in low‐resource and developing healthcare systems [25]. Although nurses find fulfillment in core clinical duties, overall motivation remains constrained by limited career advancement and extrinsic rewards.

A significant negative correlation was found between burnout and job satisfaction (*r* = −0.40, *p* < 0.001), supporting prior regional and international evidence [26–28]. This relationship aligns with COR theory [10], which posits that stress arises when vital personal resources (time, energy, emotional stability) are depleted by heavy workloads. Ongoing resource depletion triggers loss spirals that undermine mental health and job fulfillment [11]. Mitigating these resource losses requires manageable workloads, leadership support, and structured well‐being programs. Regression modeling confirmed that the nurse‐to‐patient ratio was a key predictor of burnout (β = 0.23, *p* = 0.012), with nurses caring for ≥ 3 patients experiencing significantly higher EE than those caring for 1–2 patients. This reinforces systematic review evidence linking staffing ratios directly to nurse fatigue, dissatisfaction, and turnover intent [29–31]. Furthermore, mediation analysis demonstrated that EE fully mediated the relationship between nurse‐to‐patient ratios and job satisfaction (indirect β = −0.12, Sobel *z* = −3.44, *p* < 0.001). Consistent with COR theory and emergency nursing literature [32], excessive patient assignments deplete emotional reserves, which subsequently depresses job satisfaction.

Although work unit was not a significant regression predictor, chi‐squared analysis revealed that NICU (73.1%) and ED (57.1%) nurses were significantly more likely to manage ≥ 3 patients. High volume, continuous patient turnover, and severe acuity in emergency and neonatal units increase vulnerability to EE, moral distress, and compassion fatigue [33–35]. This confirms that disproportionate workload distribution, rather than unit type per se, drives burnout risk.

Nationality also significantly predicted burnout (β = −0.23) and job satisfaction (β = 0.59). Omani nurses reported higher EE and DP, whereas non‐Omani nurses reported higher PA and job satisfaction. Prolonged exposure to local systemic stressors, cultural role expectations, and institutional responsibilities may heighten emotional strain among national nurses. In contrast, expatriate nurses may perceive local employment as a favorable professional opportunity with superior financial benefits. This pattern aligns with Gulf‐region literature documenting higher occupational stress among national nurses compared to expatriate peers [36–38].

Finally, while age, experience, and health status correlated with burnout in bivariate analyses, their effects were nonsignificant in multivariate models. Experienced nurses develop adaptive coping mechanisms over time [19, 39, 40], whereas pre‐existing health conditions exacerbate physical strain [17, 41]. However, the attenuation of these individual factors in full regression models confirms that structural work conditions—specifically nurse‐to‐patient ratios and staffing equity—exert a far dominant influence on burnout severity than personal demographics [25, 34]. Institutional strategies must therefore prioritize structural workload reforms alongside individual resilience support.

## 5. Implications for Nursing Management

### 5.1. Acuity‐Based Workload Allocation and Staffing Models

Grounded in COR theory, protecting nurses’ emotional resources through manageable workloads simultaneously mitigates EE and enhances job satisfaction, directly reflecting the observed full‐mediation mechanism. Staffing adequacy represents the primary organizational lever. Because most nurse assignments in the NICU (73.1%), ED (57.1%), and OT (51.6%) equal or exceed ≥ 1:3, nursing leaders must prioritize targeted, tiered staffing models in these high‐acuity units. Implementing flexible scheduling, task‐rotation shifts, and low‐cost coverage adjustments during peak‐acuity hours offers a feasible, evidence‐based strategy to alleviate EE without incurring substantial financial costs [30, 32].

### 5.2. Leadership Support and Peer Debriefing Protocols

Strengthened leadership support and structured professional development are essential for workplace well‐being. Nurse managers should establish peer support networks, formal debriefing protocols, and resilience workshops, which have been shown to significantly reduce work‐related stress [42]. Leadership training for nurse administrators should emphasize open communication, interdisciplinary collaboration, and shared decision‐making to cultivate a supportive practice environment [43].

### 5.3. Tailored Retention Strategies for National and Expatriate Staff

Because Omani nurses reported higher EE and non‐Omani nurses reported higher job satisfaction and PA, workforce retention policies must be differentiated. For Omani nurses, leadership should focus on workload reduction, formal local career pathways, and institutional recognition aligned with national workforce targets. For non‐Omani nurses, administrators should prioritize contract stability, supportive relocation benefits, and professional advancement opportunities. Cross‐cultural mentorship pairings and mindfulness or stress‐management programs [44] can further unify unit culture, enhance retention, and support long‐term healthcare quality.

### 5.4. Strengths and Limitations

This study presents several methodological strengths and limitations. First, sampling from a single regional hospital in Batinah South limits the generalizability of the findings to other Omani healthcare settings. The hospital operates within a geographically peripheral, secondary‐level setting characterized by resource constraints, a limited specialist workforce, reliance on expatriate nurses, and nurse‐to‐patient ratios frequently ≥ 1:3 in the NICU, ED, and OT. Cultural and professional pressures on Omani nurses, limited career mobility, and organizational constraints, including limited clinical advancement pathways, performance‐based incentives, and formal peer‐support structures, may also differ from those in better‐resourced or tertiary hospitals. Therefore, multicenter studies across different facility types and governorates are needed before drawing system‐level conclusions.

Second, reliance on self‐report instruments (MBI‐HSS [MP] and JSS) may introduce social desirability and common method bias, particularly through underreporting of DP because of professional stigma. Third, despite the high response rate (98.7%), the 1.3% nonresponse may have introduced a minor nonresponse bias if nonparticipants differed systematically from respondents in their experiences of burnout or job satisfaction. However, given the very small proportion of nonrespondents, its influence on the overall findings is likely limited. Temporary workload or unit‐specific stressors during data collection may also have influenced responses. The cross‐sectional design precludes causal inference; therefore, the mediation findings should be interpreted as statistical rather than causal. Furthermore, the regression models explained only part of the variance, suggesting that unmeasured factors such as coping styles, organizational culture, team dynamics, and leadership may also be important.

Despite these limitations, this study offers substantial empirical strengths. It addresses a critical gap in regional literature by examining burnout‐satisfaction dynamics among critical care nurses in Oman. Methodological rigor was maintained using validated instruments, stratified unit sampling, dimension‐specific reporting, verified data reanalysis, and mediation modeling. Crucially, these findings deliver actionable evidence to inform strategic nursing retention initiatives aligned with Oman Vision 2040 workforce sustainability goals.

## 6. Conclusion

This study provides key empirical evidence on the relationship between burnout and job satisfaction among critical care nurses in an Omani regional hospital. Results demonstrate moderate burnout and ambivalent job satisfaction, linked by a significant negative correlation. Crucially, EE fully mediated the pathway from nurse‐to‐patient ratios to reduced job satisfaction, offering direct support for COR theory. These findings indicate that nurse burnout is primarily driven by structural workload demands rather than individual vulnerabilities. Mitigating EE requires institutional reforms, including equitable nurse‐to‐patient ratios, improved extrinsic reward systems, and supportive leadership frameworks. Future research should utilize longitudinal designs, qualitative approaches, and multisite structural equation modeling to evaluate targeted organizational interventions across Oman’s healthcare system.

## Author Contributions

All authors contributed to study conception and design, data collection, analysis and interpretation, drafting and critical revision of the manuscript, and approved the final version for submission.

## Funding

This research received no specific grant from any funding agency in the public, commercial, or nonprofit sectors.

## Ethics Statement

Ethical approval was obtained from the Research and Ethical Review & Approval Committee in Batinah South (SBG) under the Ministry of Health (MOH) and the National Center of Health Research (Research code: 03072025, Date: July 20, 2025). The research complied with the ethical standards set by the Research and Ethical Review & Approval Committee, which are governed by a combination of national regulatory guidelines and the universal principles of the Declaration of Helsinki and World Health Organization (WHO) standards.

## Consent

Electronic informed consent was obtained from all individual participants prior to data collection via the online survey platform (Google Forms). Participants were required to read the information sheet and select a mandatory consent box before accessing the questionnaire. Participation was entirely voluntary and anonymous.

## Conflicts of Interest

The authors declare no conflicts of interest.

## Supporting Information

Additional supporting information can be found online in the Supporting Information section.

## Supporting information


**Supporting Information 1** The following supporting information is attached to this manuscript: Supporting information File (docx): Contains Supporting Tables S1–S4. Table S1: Demographic and work‐related characteristics of the critical care nurses (*N* = 150). Table S2: Descriptive statistics of burnout subscales among critical care nurses (*N* = 150). Table S3: Descriptive statistics of job satisfaction subscales among critical care nurses (*N* = 150). Table S4: Distribution of nurse‐to‐patient ratios by work unit (*N* = 150).

## Data Availability

The datasets generated and analyzed in this study are available from the corresponding author upon reasonable request.
