# Supplementary material for: Burnout Dimensions and Job Satisfaction Among Critical Care Nurses at a Regional Hospital in Oman: A Cross‐Sectional Analysis of Work‐Related Contributing Factors
Source: J Nurs Manag. 2026 Sep 7;2026:5186923. doi: 10.1155/jonm/5186923 (PMC13548049; doi:10.1155/jonm/5186923)
Supplement: Supplementary file 1 [file JONM-2026-5186923-s001.docx]

**Table S1**

*Demographic and Work-Related Characteristics of the Critical Care Nurses (N = 150)*

| Demographic Characteristics | Category | Number (*n*) | Percentage (*%*) |
| --- | --- | --- | --- |
| Gender | Male | 30 | 20.0% |
|  | Female | 120 | 80.0% |
| Nationality | Omani | 125 | 83.3% |
|  | Non-Omani | 25 | 16.7% |
| Education Level | Diploma | 53 | 35.3% |
|  | Bachelor | 91 | 60.7% |
|  | Postgraduate | 6 | 4.0% |
| Marital Status | Single | 42 | 28.0% |
|  | Married | 107 | 71.3% |
|  | Divorced | 0 | 0.0% |
|  | Widow | 1 | 0.7% |
| Health Problems | Yes | 31 | 20.7% |
|  | No | 119 | 79.3% |
| Age | ***M* = 31.83, *SD* = 6.15** | | |
| Years of experience | ***M* = 8.40, *SD* = 6.93** | | |
| Work- Related Characteristics | **Category** | **Number (*n*)** | **Percentage (*%*)** |
| Work Unit | AICU | 44 | 29.3% |
|  | PICU | 14 | 9.3% |
|  | NICU | 26 | 17.3% |
|  | ED | 35 | 23.3% |
|  | OT | 31 | 20.7% |
| Most Frequent Patient-To-Nurse Ratio | 1: 1 | 55 | 36.7% |
|  | 1: 2 | 40 | 26.7% |
|  | ≥ 1: 3 | 55 | 36.7% |
| Work Schedule | Standard Hours | 11 | 7.3% |
|  | Shift Hours | 139 | 92.7% |
| Frequency of Unscheduled Duty Requests | Never | 8 | 5.3% |
|  | Rarely (once in a while) | 43 | 28.7% |
|  | Sometimes | 80 | 53.3% |
|  | Often | 16 | 10.7% |
|  | Always | 3 | 2.0% |

**Note.** AICU = Adult Intensive Care Unit; PICU = Pediatric Intensive Care Unit; NICU = Neonatal Intensive Care Unit; OT = Operating Theatre; and ED = Emergency Department.

**Table S2**
*Descriptive Statistics of Burnout Subscales Among Critical Care Nurses (N = 150)*

| Subscale | Minimum | Maximum | *M* | *SD* |
| --- | --- | --- | --- | --- |
| EE | 0.00 | 5.67 | 2.71 | 1.36 |
| DP | 0.00 | 5.00 | 1.64 | 1.21 |
| PA | 0.75 | 6.00 | 4.35 | 1.08 |
| Total Burnout | 1.21 | 5.24 | 2.90 | 0.78 |

**Note.** *M* = Mean; *SD* = Standard Deviation; EE = Emotional Exhaustion; DP = Depersonalization; PA = Personal Accomplishment.

**Table S3**
*Descriptive Statistics of Job Satisfaction Subscales Among Critical Care Nurses (N = 150)*

| Subscale | Minimum | Maximum | *M* | *SD* |
| --- | --- | --- | --- | --- |
| Pay | 1.00 | 6.00 | 3.21 | 0.91 |
| Promotion | 1.00 | 5.25 | 3.09 | 1.02 |
| Supervision | 1.75 | 6.00 | 4.26 | 1.00 |
| Fringe Benefits | 1.00 | 5.75 | 3.03 | 0.92 |
| Contingent Reward | 1.00 | 6.00 | 3.19 | 0.87 |
| Operating Conditions | 1.00 | 4.75 | 3.15 | 0.63 |
| Coworkers | 2.25 | 6.00 | 4.06 | 0.80 |
| Nature of Work | 2.25 | 6.00 | 4.73 | 0.92 |
| Communication | 1.50 | 6.00 | 3.83 | 0.98 |
| Total JSS | 2.14 | 5.44 | 3.62 | 0.57 |

**Note.** *M* = Mean; *SD* = Standard Deviation; JSS = Job Satisfaction Survey. Higher scores indicate greater job satisfaction. Scores of 1–2.99 indicate dissatisfaction, 3–3.99 indicate ambivalence, and 4–6 indicate satisfaction.

**Table S4**

*Distribution of Nurse-to-Patient Ratios by Work Unit (N = 150)*

| Work Unit | 1:1 (%) | 1:2 (%) | ≥1:3 (%) | Total (*n*) |
| --- | --- | --- | --- | --- |
| AICU | 63.6 | 36.4 | 0.0 | 44 |
| PICU | 71.4 | 28.6 | 0.0 | 14 |
| NICU | 3.8 | 23.1 | 73.1 | 26 |
| ED | 8.6 | 34.3 | 57.1 | 35 |
| OT | 41.9 | 6.5 | 51.6 | 31 |

**Note.** Percentages represent proportions within each work unit. The chi-square test indicated a significant association between work unit and nurse-to-patient ratio, χ² (8, *N* = 150) = 72.52, *p* < .001.
